# Association between polypharmacy and the long-term prescription of hypnotics in Japan: a retrospective cross-sectional study

**DOI:** 10.3389/fpsyt.2024.1471457

**Published:** 2024-12-09

**Authors:** Munehiro Komatsu, Masahiro Takeshima, Kazuhisa Yoshizawa, Masaya Ogasawara, Mizuki Kudo, Eru Miyakoshi, Yu Itoh, Nana Shibata, Naoko Ayabe, Kazuo Mishima

**Affiliations:** ^1^ Department of Neuropsychiatry, Akita University Graduate School of Medicine, Akita, Akita, Japan; ^2^ Department of Neuropsychiatry, Akita City Hospital, Akita, Akita, Japan; ^3^ Department of Regional Studies and Humanities, Faculty of Education and Human Studies, Akita University, Akita, Akita, Japan

**Keywords:** antidepressant, antipsychotic, anxiolytic, hypnotic, polypharmacy, long term prescription

## Abstract

**Introduction:**

Hypnotic polypharmacy and its long-term prescriptions constitute the inappropriate use of hypnotics. However, the relationship between hypnotic polypharmacy and prolonged prescriptions remains unclear. This study aimed to elucidate the association between hypnotic polypharmacy and the duration of hypnotic prescriptions.

**Methods:**

This retrospective, cross-sectional study utilized a large dataset from the Japan Medical Data Center. The study population included adults who had been prescribed hypnotics between April 2020 and March 2021, with a focus on those receiving hypnotics in March 2021. Hypnotic polypharmacy was defined as the concurrent prescription of two or more hypnotics in March 2021. The duration of hypnotic prescriptions was measured by calculating the number of months between April 2019 and March 2021 during which hypnotics were prescribed. A binary logistic regression analysis was conducted to assess the relationship between hypnotic polypharmacy and long-term hypnotic prescriptions, adjusting for relevant covariates.

**Results:**

We included 112,256 patients (mean age: 49.5 years, females: 47.1%). Among them, 67.9% received hypnotic monotherapy, and 32.1% received hypnotic polypharmacy. Compared with adults who were prescribed hypnotics for 1 month, the association with polypharmacy was stronger in those who were prescribed hypnotics for ≥4 months as the duration of the prescription increased (adjusted odds ratio [aOR]: 1.15, 95% confidence interval [CI]: 1.04–1.27, p=0.006 for 4–6 months; aOR 1.35, 95% CI 1.23–1.49, p<0.001 for 7–9 months; aOR 1.58, 95% CI 1.43–1.73, p<0.001 for 10–12 months; and aOR 3.24, 95% CI 2.99–3.52 for 13–24 months).

**Conclusions:**

This study demonstrated a significant association between hypnotic polypharmacy and long-term prescriptions of hypnotics. Initiating insomnia treatment with hypnotic monotherapy may reduce the likelihood of long-term prescriptions, and limiting the duration of hypnotic prescriptions could potentially prevent polypharmacy.

## 1 Introduction

Insomnia is one of the most common sleep disorders (1). It can induce biological changes, such as disrupted sleep and restless rapid eye movement sleep, which can impair an individual’s ability to cope with emotional distress (2). Insomnia not only affects patients by causing distress (3), functional impairment (4), and reduced quality of life (5), but also has broader societal and economic consequences, including increased healthcare costs (6, 7), reduced work productivity (8–10), workplace accidents (11), and traffic accidents (12).

Cognitive behavioral therapy for insomnia (CBTi) is a multi-component psychotherapy that includes sleep restriction, stimulus control, cognitive therapy, relaxation techniques, and sleep hygiene education. CBTi has consistently been shown to be effective not only in alleviating insomnia symptoms but also in reducing insomnia-related functional impairments, such as depressed mood, anxiety, daytime sleepiness, and fatigue (13–15). Additionally, CBTi’s benefits extend beyond the treatment period, with follow-up assessments up to one year later demonstrating mild to moderate lasting effects (15). Furthermore, CBTi is considered very safe, and although daytime sleepiness and fatigue may occur in the short term following the initiation of treatment, these symptoms typically resolve by the end of treatment, and no serious side effects have been reported (16). Given its favorable risk-benefit profile, CBTi is recommended as a first-line treatment for chronic insomnia disorder in major clinical guidelines (13–15). Despite this, CBTi is underutilized due to a significant gap between availability and demand. The need for specialized training to administer CBTi, the time commitment required, and a lack of awareness among general practitioners are key barriers to its widespread use (16–18).

Monotherapy with hypnotics is recommended for treating insomnia disorder when CBTi (16), the conventional psychotherapy for insomnia is either insufficiently effective or unavailable to patients (13–15). The American Academy of Sleep Medicine provides a weak recommendation for monotherapy with certain hypnotics: ramelteon and triazolam for sleep-onset insomnia, suvorexant for sleep-maintenance insomnia, and eszopiclone and zolpidem for sleep-onset and sleep-maintenance insomnia in chronic insomnia (14).

In contrast, no guideline recommends hypnotic polypharmacy (the concomitant use of different hypnotics) for chronic insomnia disorders (13–15). This is due to the lack of evidence supporting the effectiveness of hypnotic polypharmacy and concerns that it may increase the risk of adverse effects. However, in clinical practice, hypnotic polypharmacy is frequently prescribed for patients with insomnia. Our previous study using a large claims database found that the rate of hypnotic polypharmacy among patients prescribed hypnotics increased from 18.0% in April 2005 to 22.3% in April 2019 (19). A Swedish observational study reported that among older adults prescribed benzodiazepines and benzodiazepine-related drugs (including hypnotics and anxiolytics), 19.1% were receiving polypharmacy (20). Additionally, a French cross-sectional study of nursing home residents found that 9.4% received two or more benzodiazepines (including hypnotics and anxiolytics) (21). To reduce hypnotic polypharmacy, it is crucial to identify factors associated with its use and implement interventions targeting these factors. Despite this need, very few studies have investigated the factors associated with hypnotic polypharmacy. To date, the only identified factors include the severity of depressive symptoms and a late sleep schedule (22).

The long-term prescription of hypnotics is a potentially important factor that may be associated with hypnotic polypharmacy. A retrospective cohort study using the Japanese Medical Data Center (JMDC) database reported that long-term users were more likely to be prescribed hypnotics with multiple mechanisms of action (MOA) than new users (18.2% vs. 2.8%) in 2018–2019 (22). However, this study focused on the MOA of hypnotics and did not examine the number of hypnotics independent of their MOA (22). Moreover, although the study defined long-term users of hypnotics as patients who had been prescribed the same MOA for ≥180 days, a methodological issue was that patients who had used hypnotics with different MOA for brief periods and consequently used hypnotics for extended periods were also categorized as new users. Furthermore, the correlation between the duration of hypnotic prescription and polypharmacy remains unclear, as the previous study divided patients into two groups (long-term users and new users) based on a prescription period of 180 days. To address these limitations, it is essential to investigate the relationship between the duration of hypnotic prescriptions and hypnotic polypharmacy, defining polypharmacy as the number of hypnotics prescribed and the duration of hypnotic prescription as the total period during which any hypnotics were prescribed.

Other potential factors associated with hypnotic polypharmacy include concomitant psychotropic medications and their polypharmacy. Our previous study showed that the prescription of antidepressants and antipsychotics was associated with benzodiazepine anxiolytic polypharmacy and, further, that polypharmacy for antidepressants and antipsychotics was strongly associated with benzodiazepine anxiolytic polypharmacy (23). As with anxiety, sleep disturbances and psychiatric disorders are closely related (24, 25). Patients receiving polypharmacy with antidepressants and antipsychotics may be refractory patients who cannot be managed with monotherapy, which aligns with the first recommendation in the guidelines (26, 27), or they may have been exposed to inappropriate prescribing by physicians. Consequently, these patients may also be at high risk for polypharmacy with hypnotics. Moreover, antidepressants and antipsychotics have been shown to induce sleep-related adverse effects, including insomnia and somnolence. Meta-analyses of randomized controlled trials demonstrated that compared to placebo, most antidepressants and antipsychotics were associated with a higher risk of insomnia or somnolence (28, 29). These findings have been corroborated by studies utilizing data from the Food and Drug Administration (FDA) Adverse Events Reporting System (30, 31). Given the potential for antidepressants and antipsychotics to impact sleep, it is plausible that they may be associated with hypnotic polypharmacy, either positively or negatively. However, this hypothesis has yet to be empirically tested in a real-world setting. The causes of insomnia may also be associated with hypnotic polypharmacy. These include physiological changes such as aging (1) and female gender (32), mental disorders such as depressive and anxiety disorders (15), physical disorders such as cardiovascular diseases and diabetes mellitus, neurological disorders such as neurodegenerative diseases and cerebrovascular diseases (15), and substance use such as cannabis and stimulants (15). In light of the multitude of potential causes of insomnia, a large-scale investigation is imperative to elucidate the relationship between these factors and hypnotic polypharmacy. We have conducted pharmaco-epidemiological studies utilizing the JMDC database, the largest claims database in Japan (19, 23, 33, 34). See **Supplementary Table S1** for details of our previous studies using the JMDC database (**Supplementary Table S1**). This research method is suitable for an exploratory investigation of the many factors that may be related to hypnotic polypharmacy.

Therefore, this cross-sectional study aimed to investigate whether hypnotic polypharmacy is associated with long-term prescriptions of hypnotics, concomitant psychotropic medications, and factors that cause insomnia using a large-scale claims database in Japan.

## 2 Materials and methods

### 2.1 JMDC database

The JMDC database, one of the largest public healthcare databases in Japan, includes extensive patient demographic information such as sex, age, and subscriber type (employees and their families). It also comprises medical and pharmacy claims data, clinical diagnoses based on the 10th revision of the International Statistical Classification of Diseases and Related Health Problems (ICD-10), and health check-up records that can be tracked across various hospitals and clinics within Japan. The database primarily includes employees of large private companies and their family members, ranging in age from 0 to 74 years. Since employees are typically of working age, and individuals aged ≥75 years are covered by the Advanced Elderly Medical Service System in Japan, the majority of JMDC subscribers are ≤65 years old.

### 2.2 Study design and dataset used

This retrospective cross-sectional study used claims data from the JMDC that we extracted from our previous study (33). In that study, monthly information from April 2005 to March 2021 (age, sex, subscriber type, prescription of each psychotropic medication [hypnotics, anxiolytics, antidepressants, and antipsychotics] that can be prescribed under Japan’s health insurance system, and clinical diagnoses based on the ICD-10) was extracted in June 2021 to examine the effects of Japanese policy intervention on long-term prescriptions of hypnotics. **Supplementary Table S2** provides details of the psychotropic medications extracted from the JMDC database in our previous studies (**Supplementary Table S2**). Diagnoses included 2-digit ICD-10 codes for all diseases and 3-digit ICD-10 codes for nonorganic insomnia (F51.0), nonorganic hypersomnia (F51.1), nonorganic disorder of the sleep-wake schedule (F51.2), insomnia (G47.0), hypersomnia (G47.1), circadian rhythm sleep disorders (G47.2), sleep apnea (G47.3), and narcolepsy and cataplexy (G47.4). Of the data previously extracted, data from April 2019 to March 2021 were reused in this study. The analysis was conducted between April 24 and October 30, 2024. This study was reported in accordance with the Strengthening the Reporting of Observational Studies in Epidemiology (STROBE) reporting guidelines (35).

### 2.3 Variables

#### 2.3.1 Psychotropics

Psychotropic drugs (hypnotics, anxiolytics, antidepressants, and antipsychotics) that can be prescribed under Japanese insurance were included in this study. Psychotropic drugs are classified based on Anatomical Therapeutic Chemical (ATC) codes in principle. Because barbiturates and passiflora extract are rarely used as hypnotics today and are not recommended in insomnia guidelines (14, 15), they were not considered hypnotics in this study. Although melatonin is covered by Japanese health insurance for the treatment of sleep onset difficulties associated with neurodevelopmental disorders in childhood, it is sometimes prescribed for insomnia, so in this study, we considered it to be hypnotic. Psychotropic drugs not classified by ATC were classified based on their mechanism of action and Japanese classification as follows: haloxazolam and rilmazafone are benzodiazepine hypnotics; lemborexant is an orexin receptor antagonists; flutazolam, flutoprazepam, and oxazolam are benzodiazepine anxiolytics; tandospirone is an azapirone anxiolytic; setiptiline is an antidepressant; and blonanserin, clocapramine, floropipamide, nemonapride, perospirone, spiperone, and timiperone are antipsychotics. Benzodiazepine anxiolytics before bedtime was considered hypnotics, and those prescribed during the day were considered anxiolytics. This is because benzodiazepine anxiolytics are often prescribed before bedtime to promote sleep, and the mechanism of action is considered more important than the classification (36). Similarly, hydroxyzine prescribed before bedtime was considered hypnotics, and hydroxyzine prescribed during the day was considered anxiolytics. In addition, if quetiapine or trazodone were prescribed at low doses (≤50 mg/day) only before bedtime, they were also considered hypnotics. Sulpiride is classified as an antipsychotic drug in the ATC system. According to the Japanese insurance system, sulpiride is usually administered orally at a dose of 300 mg to 600 mg per day for schizophrenia, with a maximum dose of 1200 mg/day. For depression, the dose is 150 to 300 mg per day, with a maximum of 600 mg/day. Therefore, in this study, sulpiride ≤300 mg/day was considered an antidepressant, and sulpiride >300 mg/day was considered an antipsychotic. **Table 1** provides the classification of psychotropic medications in this study based on ATC codes and Japanese insurance coverage (**Table 1**). Zaleplon and doxepin cannot be prescribed under Japanese insurance. In Japan, the sale of melatonin is prohibited in over-the-counter sales in accordance with relevant legislation. The sale of passiflora extract in Japan was discontinued in 2008.

**Table 1:** Classification of psychotropic medications in this study based on Anatomical Therapeutic Chemical (ATC) Classification codes and Japanese insurance coverage.

Hypnotics
Benzodiazepine
Alprazolam (N05BA12)*, bromazepam (N05BA08)*, brotizolam (N05CD09), chlordiazepoxide (N05BA02)*, clorazepate (N05BA05)*, clotiazepam (N05BA21)*, cloxazolam (N05BA22)*, diazepam (N05BA01)*, estazolam (N05CD04), etizolam (N05BA19)*, fludiazepam (N05BA17)*, flutazolam (none)*, flutoprazepam (none)*, flunitrazepam (N05CD03), flurazepam (N05CD01), haloxazolam (none), loflazepate (N05BA18)*, lorazepam (N05BA06)*, lormetazepam (N05CD06), medazepam (N05BA03)*, mexazolam (N05BA25)*, nimetazepam (N05CD15), nitrazepam (N05CD02), oxazolam (none)*, quazepam (N05CD10), rilmazafone (none), tofisopam (N05BA23)*, triazolam (N05CD05)
Z-drugs
Eszopiclone (N05CF04), zolpidem (N05CF02), zopiclone (N05CF01)
Melatonin receptor agonists
Melatonin (N05CH01), ramelteon (N05CH02)
Orexin receptor antagonist
Lenvorexant (none), suvorexant (N05CM19)
Antihistamine
Hydroxyzine (N05BB01)*
Antidepressants
Trazodone of (50 mg/day or less) (N06AX05)**
Antipsychotics
Quetiapine (50 mg/day or less) (N05AH04)**
Anxiolytics
Benzodiazepine
Alprazolam (N05BA12)***, bromazepam (N05BA08)***, chlordiazepoxide (N05BA02)***, clorazepate (N05BA05)***, clotiazepam (N05BA21)***, cloxazolam (N05BA22)***, diazepam (N05BA01)***, etizolam (N05BA19)***, fludiazepam (N05CD03)***, flutazolam (none)***, flutoprazepam (none)***, loflazepate (N05BA18)***, lorazepam (N05BA06)***, medazepam (N05BA03)***, mexazolam (N05BA25)***, oxazolam (none)***, tofisopam (N05BA23)***
Azapirone
Tandospirone (–)***
Antihistamine
Hydroxyzine (N05BB01)***
Antidepressants
Amitriptyline (N06AA09), amoxapine (N06AA17), clomipramine (N06AA04), dosulepin (N06AA16), duloxetine (N06AX21), escitalopram (N06AB10), fluvoxamine (N06AB08), imipramine (N06AA02), lofepramine (N06AA07), maprotiline (N06AA21), mianserin (N06AX03), milnacipran (N06AX17), mirtazapine (N06AX11), nortriptyline (N06AA10), paroxetine (N06AB05), sertraline (N06AB06), setiptiline (none), sulpiride of 300 (mg/day or less) (N05AL01), trazodone (N06AX05), trimipramine (N06AA06), venlafaxine (N06AX16), Vortioxetine (N06AX26)
Antipsychotics
Aripiprazole (N05AX12), asenapine (N05AH05), blonanserin (none), blonanserin (tape) (none), brexpiprazole (N05AX16), bromperidol (N05AD06), chlorpromazine (N05AA01), clocapramine (none), clozapine (N05AH02), floropipamide (none), fluphenazine (N05AB02), haloperidol (N05AD01), levomepromazine (N05AA02), moperone (N05AD04), mosapramine (N05AX10), nemonapride (none), olanzapine (N05AH03), oxypertine (N05AE01), paliperidone (N05AX13), perospirone (none), perphenazine (N05AB03), pimozide (N05AG02), prochlorperazine (N05AB04), quetiapine (N05AH04), risperidone (N05AX08), spiperone (none), sulpiride (> 300mg/day) (N05AL01), sultopride (N05AL02), tiapride (N05AL03), timiperone (none), zotepine (N05AX11)

The brackets indicate the Anatomical Therapeutic Chemical Classification code.

*The drug was considered a hypnotic when prescribed before bedtime.

**The drug was considered a hypnotic when prescribed at low doses (up to 50 mg/day) only before bedtime.

***The drug was considered a hypnotic when prescribed during the day.

#### 2.3.2 Diagnosis

This study included 2-digit ICD-10 codes for all diseases and 3-digit ICD-10 codes for nonorganic insomnia (F51.0), nonorganic hypersomnia (F51.1), nonorganic disorder of the sleep-wake schedule (F51.2), insomnia (G47.0), hypersomnia (G47.1), circadian rhythm sleep disorders (G47.2), sleep apnea (G47.3), and narcolepsy and cataplexy (G47.4). In this study, insomnia was defined as either F51.0 or G47.0.

### 2.4 Study population: inclusion and exclusion criteria

Patients with insomnia were eligible if they were between 20 and 74 years old and had been prescribed hypnotics in March 2021. Subscribers who had not been continuously enrolled in the JMDC database for more than 2 years as of March 2021, i.e., those who joined after May 2019, were excluded.

### 2.5 Primary outcome

The primary outcome of this study was to examine the association between hypnotic polypharmacy and the long-term prescription of hypnotics. In this study, hypnotic polypharmacy was defined as being prescribed two or more different hypnotics in March 2021. As mentioned above, hypnotics were defined using a classification system developed for this study based on the ATC code. For example, if a patient was prescribed eszopiclone and lorazepam at bedtime, the patient was considered to have been prescribed two hypnotics (i.e., hypnotic polypharmacy). The hypnotic prescription duration was defined as the number of months between April 2019 and March 2021 in which hypnotics were prescribed (for example, a patient prescribed hypnotics in July 2020 and March 2021 had a prescription duration of 2 months).

### 2.6 Secondary outcome

The secondary outcome of this study was to examine the association between hypnotic polypharmacy and psychotropic medications other than hypnotics (antidepressants, antipsychotics, benzodiazepine anxiolytics prescribed during the day, azapirone anxiolytic [tandospirone], and antihistamine [hydroxyzine]). We examined whether monotherapy or polypharmacy of each psychotropic drug was associated with hypnotic polypharmacy compared to patients who were not prescribed each psychotropic drug. Polypharmacy for psychotropic medications other than hypnotics was defined as two or more prescriptions for different drugs of the same class, as defined in this study, in March 2021. We also examined the relationship between hypnotic polypharmacy and individual antipsychotics/antidepressants.

### 2.7 Covariates

We included the following medical, mental, and neurological disorders as covariates, as they may serve as causative or contributory factors for insomnia, based on the European Insomnia Guideline 2023 (15): depressive disorders [F32, F33, F34, F38, 39], bipolar disorders [F30, F31], anxiety disorders [F40, F41], reactions to severe stress and adjustment disorders [F43], personality disorders [F60], schizophrenia [F20, F21, F22, F33, F24, F25, F28, F29], alcohol-related disorders [F10], opioid-related disorders [F11], cannabis-related disorders [F12], sedative, hypnotic, or anxiolytic-related disorders [F13], cocaine-related disorders [F14], other stimulant-related disorders, including caffeine [F15], hallucinogen-related disorders [F16], nicotine dependence [F17], inhalant-related disorders [F18], multiple drug use and other psychoactive substance-related disorders [F19], cardiovascular disorders [I21, 122, 123, 124, 125, 128, 129], diabetes mellitus [E10-E14], chronic kidney disease [N18], chronic obstructive pulmonary diseases [J43, J44], rheumatic disorders [M05, M06], chronic pain [R52], any kind of malignant disorder [O00-99], sleep-related breathing disorder (SRBD) [G47.3], neurodegenerative diseases [G10, G20, G23, G30, G31, F00, F02], cerebrovascular diseases [I60, 161], traumatic brain injury [S06, S07], and multiple sclerosis [G35]. In this study, patients with two or more diagnoses within the ICD-10 range of F11 to F18 were classified as having F19. Additionally, circadian rhythm sleep-wake disorder (CRSWD) [F51.2, G47.2] was included as a covariate, as hypnotics are often prescribed in clinical practice despite the lack of established pharmacotherapy for CRSWD (37).

### 2.8 Statistical analyses

Continuous and categorical variables were expressed as mean and standard deviation (SD) or as numbers and percentages, respectively. The chi-square test and *post-hoc* comparison z-test with Bonferroni correction were used to compare categorical variables between the hypnotic monotherapy and polypharmacy groups.

A binary logistic regression model was employed to examine the association between hypnotic polypharmacy and the long-term prescription of hypnotics (1, 2–3, 4–6, 7–9, 10–12 months, and 13–24 months), adjusting for age groups (20–39, 40–64, and 65–74 years); sex; type of subscriber (employees and family members); the number of concomitant hypnotics, antidepressants, antipsychotics, and daytime-prescribed benzodiazepine anxiolytics (categorized as zero, one, two, or more); daytime-prescribed hydroxyzine; tandospirone; and factors contributing to or causing insomnia [SRBD, CRSWD, substance use disorders (none, alcohol-related disorders, opioid-related disorders, cannabis-related disorders, sedative, hypnotic, or anxiolytic-related disorders, cocaine-related disorders, other stimulant-related disorders including caffeine, hallucinogen-related disorders, nicotine dependence, inhalant-related disorders, multiple drug use and other psychoactive substance-related disorders), schizophrenia, depressive disorders, bipolar disorders, anxiety disorders, reaction to severe stress and adjustment disorders, personality disorders, cardiovascular disorders, diabetes mellitus, chronic kidney disease, chronic obstructive pulmonary diseases, rheumatic disorders, chronic pain, any type of malignant disorder, neurodegenerative diseases, cerebrovascular diseases, traumatic brain injury, and multiple sclerosis] in model 1. In Model 2, to investigate the association between hypnotic polypharmacy and individual antidepressants/antipsychotics, we conducted a logistic regression analysis. In this model, the covariates for the number of antidepressants/antipsychotics in Model 1 were replaced with categories for antidepressants (none, individual antidepressant monotherapy, antidepressant polypharmacy) and antipsychotics (none, individual antipsychotic monotherapy, antipsychotic polypharmacy). Adjusted odds ratios (aORs), 95% confidence intervals (CIs), and associated p-values were derived from the multivariate logistic models. To ensure robustness, a sensitivity analysis was performed by excluding patients diagnosed with physical disorders, those diagnosed with mental disorders, and those prescribed psychotropic drugs other than hypnotics. All statistical analyses were conducted using SPSS Statistics version 28.0 (IBM Corp., Armonk, NY, USA). Statistical significance was defined as p < 0.05 (two-sided).

### 2.9 Ethics

This study was approved by the Ethics Committee of Akita University Graduate School of Medicine (No. 3139, date of approval: April 24, 2024). The study was conducted in accordance with the guidelines of the Declaration of Helsinki. The requirement for informed consent from patients was waived as anonymized datasets were used for analysis in this study.

## 3 Results

**Figure 1:**
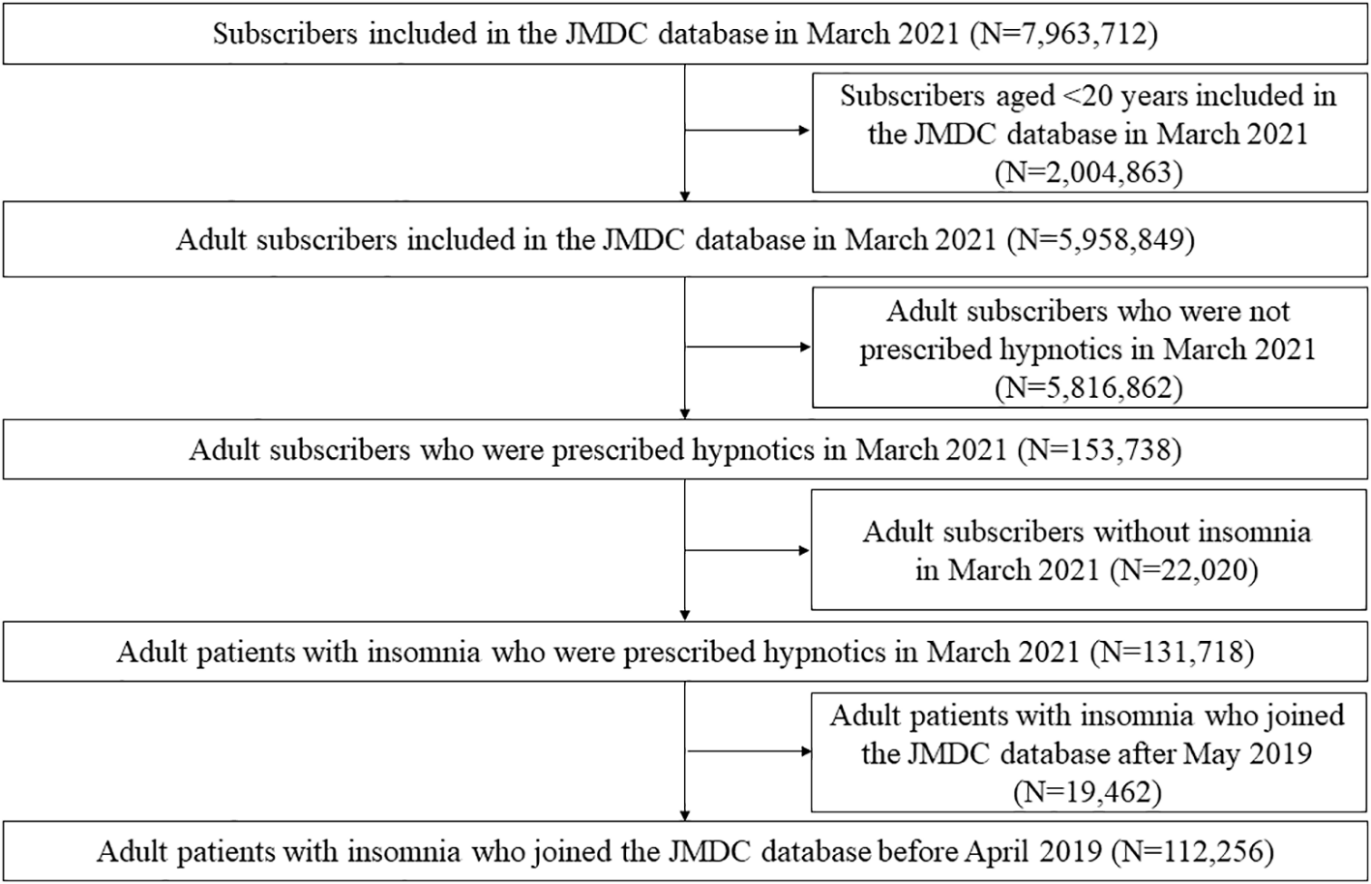
Participant selection flowchart. JMDC, Japan Medical Data Center.

**Figure 1** shows the selection of participants. In March 2021, the JMDC database contained data on 7,963,712 subscribers. Among them, 131,718 adult patients with insomnia were prescribed hypnotics. A total of 19,462 patients were excluded because they joined the JMDC after May 2019. Finally, 112,256 participants were included in the analysis. The mean (SD) age was 49.5 ± 12.2 years, and 47.1% were female. Among the patients prescribed hypnotics, 67.9% received hypnotic monotherapy, and 32.1% received hypnotic polypharmacy.

**Table 2** and **Supplementary Table S3** show the clinical and demographic characteristics of the study participants. The hypnotic polypharmacy group had a higher percentage of patients aged 20–39 and 40–64 years and a lower percentage of patients aged ≥65 years compared to the hypnotic monotherapy group. Regarding the duration of hypnotic prescription, the percentage of patients prescribed hypnotics for 13–24 months was higher in the hypnotic polypharmacy group than in the hypnotic monotherapy group (81.0 vs. 58.7%), whereas the percentage of those in other prescription-duration categories was higher in the monotherapy group than in the polypharmacy group. Compared to the hypnotic monotherapy group, the hypnotic polypharmacy group was less likely to be prescribed antidepressants overall but more likely to be prescribed antidepressant monotherapy and polypharmacy. Antipsychotic drugs followed the same pattern as antidepressants. Regarding comorbidities, the hypnotic polypharmacy group had a higher percentage of patients with comorbid psychiatric disorders, chronic pain, and cerebrovascular diseases compared with the hypnotic monotherapy group.

**Table 2:** Clinical and demographic characteristics.

	Total	Monotherapy	Polypharmacy	p-value	
	N=112256	N=76207	N=36049		
Age group				<0.001**	
20–39	23664 (21.1%)	15366 (20.2%)	8298 (23.0%)		M<P
40–64	77665 (69.2%)	52031 (68.3%)	25634 (71.1%)		M<P
>=65	10927 (9.7%)	8810 (11.6%)	2117 (5.9%)		M>P
Sex				<0.001**	
Male	59434 (52.9%)	39804 (52.2%)	19630 (54.5%)		
Female	52822 (47.1%)	36403 (47.8%)	16419 (45.5%)		
Subscriber				0.074	
Him/Herself	75794 (67.5%)	51585 (67.7%)	24209 (67.2%)		
Families	36462 (32.5%)	24622 (32.3%)	11840 (32.8%)		
Hypnotic prescription duration				<0.001**	
1 month	5341 (4.8%)	4604 (6.0%)	737 (2.0%)		M>P
2–3 months	6894 (6.1%)	5791 (7.6%)	1103 (3.1%)		M>P
4–6 months	8713 (7.8%)	7224 (9.5%)	1489 (4.1%)		M>P
7–9 months	8669 (7.7%)	7004 (9.2%)	1665 (4.6%)		M>P
10–12 months	8697 (7.7%)	6834 (9.0%)	1863 (5.2%)		M>P
13–24 months	73942 (65.9%)	44750 (58.7%)	29192 (81.0%)		M<P
Hypnotics					
BZ	62736 (55.9%)	33263 (43.6%)	29473 (81.8%)	<0.001**	
Z-drugs	49527 (44.1%)	31499 (41.3%)	18028 (50.0%)	<0.001**	
MRA	4877 (4.3%)	1888 (2.5%)	2989 (8.3%)	<0.001**	
ORA	17769 (15.8%)	8695 (11.4%)	9074 (25.2%)	<0.001**	
Trazodone	4553 (4.1%)	525 (0.7%)	4028 (11.2%)	<0.001**	
Quetiapine	1841 (1.6%)	323 (0.4%)	1518 (4.2%)	<0.001**	
Hydroxyzine	65 (0.1%)	14 (0%)	51 (0.1%)	<0.001**	
Anxiolytics					
BZ anxiolytics during the day	32153 (28.6%)	19187 (25.2%)	12966 (36%)	<0.001**	
Hydroxyzine during the day	49 (0%)	24 (0%)	25 (0.1%)	0.005*	
Tandospirone	918 (0.8%)	573 (0.8%)	345 (1%)	<0.001**	
Antidepressants				<0.001**	
0	68759 (61.3%)	50931 (66.8%)	17828 (49.5%)		M>P
1	32287 (28.8%)	19522 (25.6%)	12765 (35.4%)		M<P
≥2	11210 (10%)	5754 (7.6%)	5456 (15.1%)		M<P
Antipsychotics				<0.001**	
0	91855 (81.8%)	65578 (86.1%)	26277 (72.9%)		M>P
1	15215 (13.6%)	8195 (10.8%)	7020 (19.5%)		M<P
≥2	5186 (4.6%)	2434 (3.2%)	2752 (7.6%)		M<P
Sleep disorders					
SRBD	3118 (2.8%)	2090 (2.7%)	1028 (2.9%)	0.30	
CRSWD	94 (0.1%)	33 (0%)	61 (0.2%)	<0.001**	
Psychiatric disorders					
Substance use disorder				<0.001**	
None	110393 (98.3%)	75306 (98.8%)	35087 (97.3%)		M>P
Alcohol related disorders	1434 (1.3%)	672 (0.9%)	762 (2.1%)		M<P
Opioid-related disorders	2 (0%)	1 (0%)	1 (0%)		M=P
Cannabis-related disorders	1 (0%)	1 (0%)	0 (0%)		M=P
Sedative, hypnotic, or anxiolytic-related disorders	10 (0%)	4 (0%)	6 (0%)		M=P
Cocaine-related disorders	0 (0%)	0 (0%)	0 (0%)		
Other stimulant-related disorders including caffeine	15 (0%)	4 (0%)	11 (0%)		M<P
Hallucinogen-related disorders	0 (0%)	0 (0%)	0 (0%)		
Nicotine dependence	286 (0.3%)	172 (0.2%)	114 (0.3%)		M<P
Inhalant-related disorders	0 (0%)	0 (0%)	0 (0%)		
Multiple drug use and other psychoactive substance-related disorders	115 (0.1%)	47 (0.1%)	68 (0.2%)		M<P
Schizophrenia	20302 (18.1%)	10141 (13.3%)	10161 (28.2%)	<0.001**	
Depressive disorders	56305 (50.2%)	32149 (42.2%)	24156 (67%)	<0.001**	
Bipolar disorders	13443 (12%)	6750 (8.9%)	6693 (18.6%)	<0.001**	
Anxiety disorders	22731 (20.2%)	13840 (18.2%)	8891 (24.7%)	<0.001**	
Reaction to severe stress, and adjustment disorders	5807 (5.2%)	3710 (4.9%)	2097 (5.8%)	<0.001**	
Personality disorder	419 (0.4%)	203 (0.3%)	216 (0.6%)	<0.001**	
Physical disorders					
Cardiovascular disorders	11337 (10.1%)	8215 (10.8%)	3122 (8.7%)	<0.001**	
Diabetes	16032 (14.3%)	11379 (14.9%)	4653 (12.9%)	<0.001**	
Chronic kidney diseases	1836 (1.6%)	1332 (1.7%)	504 (1.4%)	<0.001**	
COPD	852 (0.8%)	614 (0.8%)	238 (0.7%)	0.009*	
Rheumatoid arthritis	1776 (1.6%)	1249 (1.6%)	527 (1.5%)	0.026*	
Chronic pain	3772 (3.4%)	2461 (3.2%)	1311 (3.6%)	<0.001**	
Cancer	268 (0.2%)	212 (0.3%)	56 (0.2%)	<0.001**	
Neurodegenerative diseases	2989 (2.7%)	1612 (2.1%)	1377 (3.8%)	<0.001**	
Cerebrovascular diseases	4602 (4.1%)	3446 (4.5%)	1156 (3.2%)	<0.001**	
Traumatic brain injury	108 (0.1%)	75 (0.1%)	33 (0.1%)	0.73	
Multiple sclerosis	114 (0.1%)	84 (0.1%)	30 (0.1%)	0.19	

Values are presented as numbers (%). P-values with significant results (<0.05) are labeled with an asterisk (*), and those with significant results (<0.001) are labeled with a double asterisk (**).

M > P indicates a significantly larger proportion of patients in the polypharmacy group than in the monotherapy group for that item, based on *post-hoc* analysis.

M < P indicates a significantly larger proportion of patients in the monotherapy group than in the polypharmacy group for that item, based on *post-hoc* analysis.

BZ, benzodiazepine; CI, confidence interval; COPD, chronic obstructive pulmonary disease; CRSWD, circadian rhythm sleep-wake disorder; MRA, melatonin receptor agonist; OR, odds ratio; ORA, orexin receptor antagonist; SRBD, sleep related breathing disorders.

**Table 3** shows the results of the logistic regression analysis. No association was observed between hypnotic polypharmacy and patients prescribed hypnotics for 1 or 2–3 months. However, the association between polypharmacy and the duration of hypnotic prescription became stronger with longer prescription durations, particularly for prescriptions of ≥4 months (aOR: 1.15, 95% CI: 1.04–1.27, p<0.006 for 4–6 months; aOR 1.35, 95% CI 1.23–1.49, p<0.001 for 7–9 months; aOR 1.58, 95% CI 1.43–1.73, p<0.001 for 10–12 months; and aOR: 3.24, 95% CI 2.99–3.52, p<0.001 for 13–24 months). Sensitivity analysis confirmed that there was an association between hypnotic polypharmacy and hypnotic prescriptions lasting 10–12 months and prescriptions lasting 13 months or longer. Patients who were not prescribed psychotropic medications other than hypnotics were associated with a prescription duration of more than 7 months; patients who were not diagnosed with a mental disorder were associated with a prescription duration of more than 2 months; and patients who were not diagnosed with a physical disorder were associated with a prescription duration of more than 10 months (**Supplementary Table S4**). Compared with patients who were not prescribed each type of psychotropic medication, hypnotic polypharmacy was associated with antipsychotic monotherapy and polypharmacy (aOR 1.10, 95% CI 1.05–1.15, p<0.001 and aOR 1.39, 95% CI 1.29–1.49, p<0.001, respectively), antidepressant polypharmacy (aOR 1.21, 95% CI 1.16–1.28, p<0.001), and benzodiazepine anxiolytic monotherapy (aOR 1.17, 95% CI 1.13–1.21, p<0.001). However, hypnotic polypharmacy was not associated with benzodiazepine anxiolytic polypharmacy (aOR 0.95, 95% CI 0.89–1.03, p=0.20). In addition, hypnotic polypharmacy was negatively associated with older age (aOR: 0.67, 95% CI: 0.63–0.71, p<0.001) and female sex (aOR: 0.92, 95% CI: 0.89–0.96, p<0.001). Compared with those not diagnosed with substance use disorders, hypnotic polypharmacy was positively associated with all substance use disorders except for opioid use disorder and cannabis use disorder.

**Table 3:** Factors associated with hypnotic polypharmacy.

	Adjusted OR (95% CI)	p-value
Age group		
20–39	1 [Reference]	
40–64	0.95 (0.92–0.983)	0.003*
>=65	0.67 (0.63–0.71)	<0.001**
Sex		
Male	1 [Reference]	
Female	0.92 (0.89–0.96)	<0.001**
Subscriber		
Him/Herself	1 [Reference]	
Families	0.96 (0.92–0.99)	0.016*
Hypnotic prescription duration		
1 month	1 [Reference]	
2–3 months	1.09 (0.98–1.21)	0.11
4–6 months	1.15 (1.04–1.27)	0.006*
7–9 months	1.35 (1.23–1.49)	<0.001**
10–12 months	1.58 (1.43–1.73)	<0.001**
13–24 months	3.24 (2.99–3.52)	<0.001**
Concomitant psychotropics		
Antidepressant		
0	1 [Reference]	
1	0.96 (0.92–0.99)	0.023*
≥2	1.21 (1.16–1.28)	<0.001**
Antipsychotics		
0	1 [Reference]	
1	1.10 (1.05–1.15)	<0.001**
≥2	1.39 (1.29–1.49)	<0.001**
BZ anxiolytics during the day		
0	1 [Reference]	<0.001**
1	1.17 (1.13–1.21)	<0.001**
≥2	0.95 (0.89–1.03)	0.20
Hydroxyzine during the day	1.93 (1.04–3.56)	0.036*
Tandospirone	0.98 (0.85–1.13)	0.74
Sleep disorders		
SRBD	1.00 (0.92–1.08)	0.92
CRSWD	3.79 (2.40–5.98)	<0.001**
Psychiatric disorders		
Substance use disorder		
None	1 [Reference]	<0.001**
Alcohol-related disorders	1.72 (1.538–1.92)	<0.001**
Opioid-related disorders	0.79 (0.049–12.7)	0.867
Cannabis-related disorders	Cannot be calculated***	
Sedative, hypnotic, or anxiolytic-related disorders	1.47 (0.39–5.60)	0.57
Other stimulant-related disorders including caffeine	4.29 (1.30–14.1)	0.017*
Nicotine dependence	1.31 (1.01–1.69)	0.039*
Multiple drug use and other psychoactive substance-related disorders	1.66 (1.13–2.46)	0.01*
Schizophrenia	1.55 (1.48–1.61)	<0.001**
Depressive disorders	2.16 (2.08–2.24)	<0.001**
Bipolar disorders	1.40 (1.34–1.46)	<0.001**
Anxiety disorders	1.34 (1.30–1.39)	<0.001**
Reaction to severe stress, and adjustment disorders	1.13 (1.06–1.20)	<0.001**
Personality disorder	1.23 (1.01–1.51)	0.043*
Physical disorders		
Cardiovascular disorders	0.98 (0.93–1.03)	0.32
Diabetes	0.96 (0.92–0.99)	0.044*
Chronic kidney diseases	1.07 (0.96–1.20)	0.21
COPD	1.06 (0.91–1.25)	0.46
Rheumatoid arthritis	1.06 (0.95–1.19)	0.29
Chronic pain	1.22 (1.13–1.31)	<0.001**
Cancer	0.70 (0.51–0.97)	0.031*
Neurodegenerative diseases	1.03 (0.95–1.12)	0.42
Cerebrovascular diseases	0.92 (0.85–0.99)	0.018*
Traumatic brain injury	0.98 (0.62–1.53)	0.92
Multiple sclerosis	0.87 (0.56–1.35)	0.53

P-values with significant results (<0.05) are labeled with an asterisk (*), and those with significant results (<0.001) are labeled with a double asterisk (**). The larger the odds ratio (OR), the stronger the positive association with hypnotic polypharmacy.

***The odds ratio could not be calculated because there were no patients with cannabis use disorder in the hypnotic polypharmacy group.

CI, confidence interval; COPD, chronic obstructive pulmonary disease; CRSWD, circadian rhythm sleep-wake disorder; OR, odds ratio; SRBD, sleep related breathing disorders.

**Table 4** and **Supplementary Table S5** present the results of the logistic regression analysis examining the association between hypnotic polypharmacy and individual antidepressants/antipsychotics. Compared with patients who were not prescribed antidepressants, hypnotic polypharmacy was positively associated with mianserin (aOR 1.64, 95% CI 1.23–2.19, p<0.001), amoxapine (aOR 1.41, 95% CI 1.18–1.69, p<0.001), and trazodone (aOR 1.24, 95% CI 1.09–1.40, p<0.001), and negatively associated with sertraline (aOR 0.90, 95% CI 0.84–0.96, p=0.002), clomipramine (aOR 0.79, 95% CI 0.63–0.98, p=0.033), escitalopram (aOR 0.87, 95% CI 0.81–0.93, p<0.001), and paroxetine (aOR 0.82, 95% CI 0.76–0.89, p<0.001). Compared with patients who were not prescribed antipsychotics, hypnotic polypharmacy was positively associated with tiapride (aOR 2.33, 95% CI 1.29–4.21, p=0.005), levomepromazine (aOR 2.06, 95% CI 1.79–2.37, p<0.001), chlorpromazine (aOR 1.76, 95% CI 1.49–2.07, p<0.001), sulpiride (aOR 1.54, 95% CI 1.11–2.15, p=0.01), and lurasidone (aOR 1.23, 95% CI 1.01–1.50, p=0.04), and negatively associated with olanzapine (aOR 0.85, 95% CI 0.77–0.94, p=0.001).

**Table 4:** Individual antidepressants and antipsychotics significantly associated with hypnotic polypharmacy.

	Adjusted OR (95% CI)	p-value
Antidepressants		
None	1 [Reference]	
Mianserin	1.64 (1.23–2.19)	<0.001**
Amoxapine	1.41 (1.18–1.69)	<0.001**
Trazodone	1.24 (1.09–1.40)	<0.001**
Sertraline	0.90 (0.84–0.96)	0.002*
Clomipramine	0.79 (0.63–0.98)	0.033*
Escitalopram	0.87 (0.81–0.93)	<0.001**
Paroxetine	0.82 (0.76–0.89)	<0.001**
2 or more	1.21 (1.15–1.27)	<0.001**
Antipsychotics		
None	1 [Reference]	
Tiapride	2.33 (1.29–4.21)	0.005*
Levomepromazine	2.06 (1.79–2.37)	<0.001**
Chlorpromazine	1.76 (1.49–2.07)	<0.001**
Sulpiride	1.54 (1.11–2.15)	0.01*
Lurasidone	1.23 (1.01–1.50)	0.04*
Olanzapine	0.85 (0.77–0.94)	0.001*
2 or more	1.35 (1.25–1.45)	<0.001**

P-values with significant results (<0.05) are labeled with an asterisk (*), and those with significant results (<0.001) are labeled with a double asterisk (**). The larger the odds ratio (OR), the stronger the positive association with hypnotic polypharmacy.

Adjusted for age groups (20–39, 40–64, and 65–74 years); sex; type of subscriber (employees and their family members); hypnotic prescription duration (1, 2–3, 4–6, 7–9, 10–12, and 13–24 months); the number of concomitant antidepressants, antipsychotics, benzodiazepine anxiolytics during the day, hydroxyzine during the day, and tandospirone (0, 1, 2 or more); each sleep disorder, each psychiatric disorder, and each physical disorder.

CI, confidence interval; OR, odds ratio.

## 4 Discussion

To our knowledge, this is the first study to examine the association between polypharmacy and long-term prescriptions of hypnotics. As hypothesized, hypnotic polypharmacy was associated with the duration of hypnotic prescriptions. Furthermore, hypnotic polypharmacy was associated with the polypharmacy of antidepressants and antipsychotics, as well as with certain individual antidepressants and antipsychotics, mental disorders, and some physical disorders.

Compared with patients who were prescribed hypnotics for only 1 month in the previous year, this study demonstrated that a prescription duration of ≥4 months was significantly associated with hypnotic polypharmacy, and the strength of this association increased with longer prescription durations. However, previous studies have indicated that higher doses of hypnotics at the time of initial prescription are associated with longer hypnotic prescriptions (hypnotics defined as benzodiazepines, Z-drugs, and barbiturates) (38) and that the risk of long-term hypnotic prescriptions increases with the number of hypnotics prescribed in the first month (hypnotics defined as benzodiazepines, Z-drugs, melatonin receptor agonists, orexin receptor antagonists, barbiturates, and passiflora extract) (33). Although the definitions of hypnotics differ between this study and previous studies (33, 38), and the causal relationship between hypnotic polypharmacy and longer hypnotic prescriptions remains unclear due to the cross-sectional design of this study, it is possible that polypharmacy and long-term hypnotic prescriptions have a bidirectional relationship. Accordingly, long-term prescriptions of hypnotics can potentially be reduced by initiating drug therapy for insomnia with hypnotic monotherapy, and hypnotic polypharmacy may be minimized by limiting the duration of hypnotic prescriptions. Additionally, hypnotic response must be considered when interpreting the results of this study. Although this study lacks information regarding whether patients had hypnotic-resistant insomnia, such cases could lead to prolonged hypnotic prescriptions, thereby contributing to hypnotic polypharmacy. This phenomenon is likely similar for other chronic conditions, such as diabetes, hypertension, and pain, where polypharmacy tends to increase as treatment duration extends. Previous studies have demonstrated that CBTi is effective in treating pharmacotherapy-resistant insomnia (39). Therefore, implementing CBTi in cases of pharmacotherapy-resistant insomnia may not only improve insomnia symptoms but also reduce polypharmacy and the long-term use of hypnotics.

This study found that hypnotic polypharmacy was associated with antidepressant polypharmacy and antipsychotic polypharmacy. Interestingly, these results are similar to those of our previous study, which found that anxiolytic polypharmacy was associated with antidepressant polypharmacy and antipsychotic polypharmacy (23). The reason for these findings remains unclear, as this study lacks data beyond claims information. However, one potential factor influencing the results may be the prescribing behavior of physicians: those who prefer prescribing hypnotic polypharmacy may also be inclined to prescribe antidepressant or antipsychotic polypharmacy. A study examining the impact of guideline training on Japanese physicians revealed that those who attended training exhibited higher rates of monotherapy with antipsychotics without co-prescribing other psychotropic medications for schizophrenia and higher rates of monotherapy with antidepressants for major depressive disorder compared to physicians who did not attend the training (40). Additionally, these trained physicians demonstrated lower rates of prescribing hypnotics for schizophrenia and major depressive disorder (40). Therefore, providing medical practitioners with training on clinical guidelines for mental disorders may facilitate the appropriate utilization of hypnotics.

For individual antidepressants, this study revealed that mianserin, amoxapine, and trazodone were associated with hypnotic polypharmacy. Among antidepressants available in Japan, a previous study using the FDA Adverse Events Reporting System found that amoxapine was most strongly associated with the adverse event of sleepiness, with mianserin ranking third (30). For individual antipsychotics, this study showed that tiapride, chlorpromazine, levomepromazine, sulpiride, and lurasidone were associated with hypnotic polypharmacy. Among antipsychotics available in Japan, a similar study based on the FDA Adverse Events Reporting System reported that tiapride was most strongly associated with the adverse event of sleepiness, with chlorpromazine ranking third (31). Given that psychotropic medications inducing daytime somnolence may promote sleep, physicians may have prescribed sedative psychotropic drugs to patients whose insomnia symptoms did not improve despite the use of hypnotic polypharmacy.

This study found that alcohol-related disorders, other stimulant-related disorders, including caffeine and nicotine dependence, and other psychoactive substance-related disorders were associated with hypnotic polypharmacy. Alcohol, caffeine, and nicotine are known to cause insomnia (15, 41), and withdrawal from these substances can also trigger insomnia (41). Although this study lacks detailed information on sub-diagnoses such as abuse, dependence, and withdrawal, it is understandable that there is a link between these substance use disorders and hypnotic polypharmacy. In contrast, this study found no association between hypnotic polypharmacy and opioid-related disorders, cannabis-related disorders, or sedative, hypnotic, or anxiolytic-related disorders. This lack of association may be due to insufficient statistical power, as indicated by the wide 95% confidence intervals.

In this study, comorbidities that showed a positive association with hypnotic polypharmacy included CRSWD, all psychiatric disorders, chronic pain, and cerebrovascular diseases, with particularly high odds ratios for CRSWD and depressive disorder. A Japanese internet survey of individuals with subjective insomnia reported that the severity of depressive symptoms and a late sleep schedule were associated with hypnotic polypharmacy (42), and the findings of this study are consistent with those results. Although melatonin is weakly recommended for delayed sleep-wake phase disorder with or without major depressive disorder in clinical guidelines, no other substances, hypnotics, or their combinations are recommended for intrinsic CRSWD (43). As bright light therapy has been shown to be effective in treating some forms of CRSWD (43, 44), appropriate non-pharmacological treatments may help prevent hypnotic polypharmacy.

This study found that older adults and women were negatively associated with hypnotic polypharmacy. These findings are intriguing, given that most studies identify female gender and age as risk factors for insomnia (45) and hypnotic prescriptions (46). One possible explanation for this result is that hypnotic polypharmacy increases the risk of adverse events compared to hypnotic monotherapy. Previous studies have identified polypharmacy (47), aging (48), and female gender (49) as risk factors for adverse drug reactions. Therefore, prescribers may have avoided prescribing hypnotic polypharmacy in female and elderly patients, who are at higher risk for adverse drug reactions, to minimize potential harm.

This study had several limitations. First, the JMDC database is not representative of the Japanese population, as it excludes individuals aged over 75 years and consists primarily of employees of large companies and their families. Therefore, the results of this study need to be validated using a national database. Second, since this study utilized Japanese medical fee data, certain psychotropic drugs, such as doxepin and zaleplon, were not included. Consequently, the findings may not be generalizable to other countries. Third, the claim database is primarily used for medical reimbursement purposes and was not originally designed for objective observation of clinical outcomes. Fourth, as CBTi is not covered by Japan’s insurance, this study lacked data on whether or not CBTi was performed. However, because previous studies suggest that CBTi is rarely used for insomnia in Japan (18), the lack of information on CBTi is unlikely to have affected the results. Fifth, this study lacked information on clinical symptoms, such as insomnia severity, physicians’ attitudes toward hypnotic prescription and discontinuation (50, 51), and patient treatment preferences (52). Additionally, although the claims data used in the JMDC database include diagnoses for substance use disorders, it lacks information on actual substance use. As substance use can contribute to insomnia, the absence of this data may have influenced the results (15, 41), potentially affecting the observed associations with hypnotic polypharmacy. Sixth, as this was a cross-sectional study, it was not possible to examine the processes that led to hypnotic polypharmacy. The use of certain classes of hypnotics or specific combinations of these drugs may be associated with an increased risk of hypnotic polypharmacy. Further research is required to clarify this issue. Seventh, this study lacked data on the number of hypnotics prescribed at the time of the initial prescription, making it impossible to determine whether patients who began treatment with a single hypnotic had a lower risk of developing polypharmacy compared to those who started with hypnotic polypharmacy. Eighth, this study used claims data collected during the COVID-19 pandemic. There is a possibility that the pandemic increased the number of patients with insomnia or worsened the severity of their insomnia (53). Additionally, patients with insomnia may have avoided hospital visits due to concerns about infection (54), which could have influenced the study’s results. Finally, this study did not account for potential drug interactions involving cytochrome P450 enzymes. The blood concentration of a hypnotic may fluctuate due to drug interactions with other concomitant medications. Therefore, drug-drug interactions involving cytochrome P450 enzymes may have affected the findings.

In conclusion, this study demonstrated an association between hypnotic polypharmacy and a hypnotic prescription duration of ≥4 months over 2 years, compared to 1 month over 2 years. Furthermore, the study found that the association with hypnotic polypharmacy became stronger as the duration of the prescription increased. Initiating insomnia treatment with hypnotic monotherapy may help prevent long-term hypnotic prescriptions, and minimizing the duration of hypnotic prescriptions could reduce the risk of polypharmacy. However, as this was a cross-sectional study, causal relationships could not be established, and further prospective cohort studies are required.

## Data Availability

The datasets presented in this article are not readily available. The data is available from the corresponding author upon reasonable request and with permission from JMDC.
