# Supplementary material for: Association between polypharmacy and the long-term prescription of hypnotics in Japan: a retrospective cross-sectional study
Source: Front Psychiatry. 2024 Dec 9;15:1471457. doi: 10.3389/fpsyt.2024.1471457 (PMC11663738; doi:10.3389/fpsyt.2024.1471457)
Supplement: Supplementary file 1 [file DataSheet1.pdf]

Table S1. Details of our previous studies using Japanese Medical Data Center database

No	Study	Aim	Design	Period of analyzed data	Extracted data items	Analyzed data items
1	Takeshima 2022	To examine changes in psychotropic polypharmacy and high-potency prescription following policy intervention	Cross-sectional	April 2005 to June 2019	Age, sex, type of subscriber, psychotropic medications (hypnotics, anxiolytics, antidepressants, antipsychotics)	Age, sex, psychotropic medications (hypnotics, anxiolytics, antidepressants, antipsychotics)
2	Takeshima 2023	To examine the effects of Japanese policies and novel hypnotics on long-term prescriptions of hypnotics	Cohort	April 2005 to March 2021	Age, sex, type of subscriber, diagnosis based on ICD-10, psychotropic medications (hypnotics, anxiolytics, antidepressants, antipsychotics)	Age, sex, psychotropic medications (hypnotics, anxiolytics, antidepressants, antipsychotics)
3	Takeshima 2024[a]	To determine which guideline-recommended hypnotics have lower risks of monotherapy failure and which hypnotics have a higher risk of long-term prescription for insomnia treatment.	Cohort	April 2005 to March 2021	Not applicable (Secondary analysis of Takeshima 2023)	Age, sex, type of subscriber, diagnosis based on ICD-10, psychotropic medications (hypnotics, anxiolytics, antidepressants, antipsychotics)

4	Takeshima 2024[b]	To examine the association between benzodiazepine anxiolytic polypharmacy and concomitant psychotropic medications	Cross sectional	June 2019	Not applicable (Secondary analysis of Takeshima 2022)	Age, sex, type of subscriber, psychotropic medications (hypnotics, anxiolytics, antidepressants, antipsychotics)
5	Present study	To examine the association between polypharmacy and the long-term prescription duration of hypnotics	Cross sectional	April 2020 to March 2021	Not applicable (Secondary analysis of Takeshima 2023)	Age, sex, type of subscriber, diagnosis based on ICD-10, psychotropic medications (hypnotics, anxiolytics, antidepressants, antipsychotics)

Note: The diagnosis included 2-digit ICD-10 codes for all diseases and 3-digit ICD-10 codes for nonorganic insomnia (F51.0), nonorganic hypersomnia (F51.1), nonorganic disorder of the sleep-wake schedule (F51.2), insomnia (G47.0), hypersomnia (G47.1), circadian rhythm sleep disorders (G47.2), sleep apnea (G47.3), and narcolepsy and cataplexy (G47.4).

Abbreviations: ICD-10, the International Statistical Classification of Diseases and Related Health Problems

Study No 1. Takeshima M, Enomoto M, Ogasawara M, Kudo M, Itoh Y, Yoshizawa K, et al. Changes in psychotropic polypharmacy and high-potency prescription following policy change: Findings from a largescale Japanese claims database. Psychiatry and clinical neurosciences. 2022.

Study No 2. Takeshima M, Yoshizawa K, Enomoto M, Ogasawara M, Kudo M, Itoh Y, et al. Effects of Japanese policies and novel hypnotics on long-term prescriptions of hypnotics. Psychiatry and clinical neurosciences. 2023;77(5):264-72.

Study No 3. Takeshima M, Yoshizawa K, Ogasawara M, Kudo M, Itoh Y, Ayabe N, et al. Treatment Failure and Long-Term Prescription Risk for

Guideline-Recommended Hypnotics in Japan. JAMA Network Open. 2024;7(4):e246865.

Study No 4. Takeshima M, Yoshizawa K, Ogasawara M, Kudo M, Itoh Y, Ayabe N, et al. Association between benzodiazepine anxiolytic polypharmacy and concomitant psychotropic medications in Japan: a retrospective cross-sectional study. Frontiers in Psychiatry. 2024;15.
