# Supplementary material for: Association between polypharmacy and the long-term prescription of hypnotics in Japan: a retrospective cross-sectional study
Source: Front Psychiatry. 2024 Dec 9;15:1471457. doi: 10.3389/fpsyt.2024.1471457 (PMC11663738; doi:10.3389/fpsyt.2024.1471457)
Supplement: Supplementary file 2 [file DataSheet2.pdf]

Table S2. List of psychotropic drugs extracted from the JMDC database in our previous study

Hypnotics

Generic name	ATC code	Sales		Data extraction	
		Release	Discontinued	Takeshima 2022[A]	Takeshima 2023[B]
Benzodiazepine					
Brotizolam	N05CD09	Prior to 2005	On sale	✓	✓
Estazolam	N05CD04	Prior to 2005	On sale	✓	✓
Etizolam	N05BA19	Prior to 2005	On sale	✓	✓
Flunitrazepam	N05CD03	Prior to 2005	On sale	✓	✓
Flurazepam	N05CD01	Prior to 2005	On sale	✓	✓
Haloxazolam	None	Prior to 2005	On sale	✓	✓
Lormetazepam	N05CD06	Prior to 2005	On sale	✓	✓
Nimetazepam	N05CD15	Prior to 2005	On sale	✓	✓
Nitrazepam	N05CD02	Prior to 2005	On sale	✓	✓
Quazepam	N05CD10	Prior to 2005	On sale	✓	✓
Rilmazafone	None	Prior to 2005	On sale	✓	✓
Triazolam	N05CD05	Prior to 2005	On sale	✓	✓
Non-benzodiazepine					
Eszopiclone	N05CF04	April 2012	On sale	✓	✓
Zolpidem	N05CF02	Prior to 2005	On sale	✓	✓
Zopiclone	N05CF01	Prior to 2005	On sale	✓	✓

Barbituric acid					
Amobarbital	N05CA02	Prior to 2005	On sale	✓	✓
Barbital	N05CA04	Prior to 2005	On sale	✓	✓
Bromovalerylurea	None	Prior to 2005	On sale	✓	✓
Pentobarbital calcium	N05CA01	Prior to 2005	On sale	✓	✓
Melatonin receptor agonists					
Melatonin[C]	N05CH01	June 2020	On sale		✓
Ramelteon	N05CH02	July 2010	On sale	✓	✓
Orexin receptor antagonist					
Lenvorexant	None	June 2020	On sale		✓
Suvorexant	N05CM19	November 2014	On sale	✓	✓
Others					
Butoctamide	None	Prior to 2005	March 2015	✓	✓
Chloral hydrate	N05CC01	Prior to 2005	On sale	✓	✓
Passiflora extract	None	Prior to 2005	March 2009	✓	✓

Note:

[A] Takeshima M, Enomoto M, Ogasawara M, Kudo M, Itoh Y, Yoshizawa K, et al. Changes in psychotropic polypharmacy and high-potency prescription following policy change: Findings from a large scale Japanese claims database. Psychiatry and clinical neurosciences. 2022.

[B] Takeshima M, Yoshizawa K, Enomoto M, Ogasawara M, Kudo M, Itoh Y, et al. Effects of Japanese policies and novel hypnotics on long-term prescriptions

of hypnotics. Psychiatry and clinical neurosciences. 2023;77(5):264-72.

[C] Insurance coverage is available for difficulty falling asleep associated with childhood neurodevelopmental disorders.

Abbreviation: ATC, the Anatomical Therapeutic Chemical classification 2021

Anxiolytics

Generic name	ATC code	Sales		Data extraction	
		Release	Discontinued	Takeshima 2022[A]	Takeshima 2023[B]
Benzodiazepine					
Alprazolam	N05BA12	Prior to 2005	On sale	✓	✓
Bromazepam	N05BA08	Prior to 2005	On sale	✓	✓
Chlordiazepoxide	N05BA02	Prior to 2005	On sale	✓	✓
Clorazepate	N05BA05	Prior to 2005	On sale	✓	✓
Clotiazepam	N05BA21	Prior to 2005	On sale	✓	✓
Cloxazolam	N05BA22	Prior to 2005	On sale	✓	✓
Diazepam	N05BA01	Prior to 2005	On sale	✓	✓
Etizolam	N05BA19	Prior to 2005	On sale	✓	✓
Fludiazepam	N05BA17	Prior to 2005	On sale	✓	✓
Flutazolam	None	Prior to 2005	On sale	✓	✓
Flutoprazepam	None	Prior to 2005	On sale	✓	✓
Loflazepate	N05BA18	Prior to 2005	On sale	✓	✓
Lorazepam	N05BA06	Prior to 2005	On sale	✓	✓
Medazepam	N05BA03	Prior to 2005	On sale	✓	✓
Mexazolam	N05BA25	Prior to 2005	On sale	✓	✓
Oxazepam	N05BA04	Prior to 2005	Prior to 2005	✓	✓
Oxazolam	None	Prior to 2005	On sale	✓	✓
Prazepam	N05BA11	Prior to 2005	March 2012	✓	✓
Tofisopam	N05BA23	Prior to 2005	On sale	✓	✓

Azapirone					
Tandospirone	–	Prior to 2005	On sale	✓	✓
Antihistamine					
Hydroxyzine	N05BB01	Prior to 2005	On sale	✓	✓

Note:

[A] Takeshima M, Enomoto M, Ogasawara M, Kudo M, Itoh Y, Yoshizawa K, et al. Changes in psychotropic polypharmacy and high-potency prescription following policy change: Findings from a large scale Japanese claims database. Psychiatry and clinical neurosciences. 2022.

[B] Takeshima M, Yoshizawa K, Enomoto M, Ogasawara M, Kudo M, Itoh Y, et al. Effects of Japanese policies and novel hypnotics on long-term prescriptions of hypnotics. Psychiatry and clinical neurosciences. 2023;77(5):264-72.

Abbreviation: ATC, the Anatomical Therapeutic Chemical classification 2021

Antidepressants

Generic name	ATC code	Sales		Data extraction	
		Release	Discontinued	Takeshima 2022[A]	Takeshima 2023[B]
Amitriptyline	N06AA09	Prior to 2005	On sale	✓	✓
Amoxapine	N06AA17	Prior to 2005	On sale	✓	✓
Clomipramine	N06AA04	Prior to 2005	On sale	✓	✓
Desipramine	N06AA01	Prior to 2005	Prior to 2005	✓	✓
Dosulepin	N06AA16	Prior to 2005	On sale	✓	✓
Duloxetine	N06AX21	April 2010	On sale	✓	✓
Escitalopram	N06AB10	August 2011	On sale	✓	✓
Fluvoxamine	N06AB08	Prior to 2005	On sale	✓	✓
Imipramine	N06AA02	Prior to 2005	On sale	✓	✓
Lofepramine	N06AA07	Prior to 2005	On sale	✓	✓
Maprotiline	N06AA21	Prior to 2005	On sale	✓	✓
Mianserin	N06AX03	Prior to 2005	On sale	✓	✓
Milnacipran	N06AX17	Prior to 2005	On sale	✓	✓
Mirtazapine	N06AX11	July 2009	On sale	✓	✓
Nortriptyline	N06AA10	Prior to 2005	On sale	✓	✓
Paroxetine	N06AB05	Prior to 2005	On sale	✓	✓
Safrazine	None	Prior to 2005	Prior to 2005	✓	✓
Sertraline	N06AB06	July 2006	On sale	✓	✓
Setiptiline	None	Prior to 2005	On sale	✓	✓
Sulpiride	N05AL01	Prior to 2005	On sale	✓	✓

Trazodone	N06AX05	Prior to 2005	On sale	✓	✓
Trimipramine	N06AA06	Prior to 2005	On sale	✓	✓
Venlafaxine	N06AX16	December 2015	On sale	✓	✓
Vortioxetine	N06AX26	November 2019	On sale		✓

Abbreviation: ATC, the Anatomical Therapeutic Chemical classification 2021

[A] Takeshima M, Enomoto M, Ogasawara M, Kudo M, Itoh Y, Yoshizawa K, et al. Changes in psychotropic polypharmacy and high-potency prescription following policy change: Findings from a large scale Japanese claims database. Psychiatry and clinical neurosciences. 2022.

[B] Takeshima M, Yoshizawa K, Enomoto M, Ogasawara M, Kudo M, Itoh Y, et al. Effects of Japanese policies and novel hypnotics on long-term prescriptions of hypnotics. Psychiatry and clinical neurosciences. 2023;77(5):264-72.

Abbreviation: ATC, the Anatomical Therapeutic Chemical classification 2021

Antipsychotics

Generic name	ATC code	Sales		Data extraction	
		Release	Discontinued	Takeshima 2022[A]	Takeshima 2023[B]
Aripiprazole	N05AX12	May 2015	On sale	✓	✓
Asenapine	N05AH05	May 2016	On sale	✓	✓
Blonanserin	None	April 2008	On sale	✓	✓
Blonanserin (tape)	None	September 2019	On sale		✓
Brexpiprazole	N05AX16	April 2018	On sale	✓	✓
Bromperidol	N05AD06	Prior to 2005	On sale	✓	✓
Carpipramine	None	August 2011	June 2013	✓	✓
Chlorpromazine	N05AA01	Prior to 2005	On sale	✓	✓
Clocapramine	None	Prior to 2005	On sale	✓	✓
Clozapine	N05AH02	July 2009	On sale	✓	✓
Floropipamide	None	Prior to 2005	On sale	✓	✓
Fluphenazine	N05AB02	Prior to 2005	On sale	✓	✓
Haloperidol	N05AD01	Prior to 2005	On sale	✓	✓
Levomepromazine	N05AA02	Prior to 2005	On sale	✓	✓
Lurasidone	N05AE05	June 2020	On sale		✓
Moperone	N05AD04	Prior to 2005	On sale	✓	✓
Mosapramine	N05AX10	Prior to 2005	On sale	✓	✓
Nemonapride	None	Prior to 2005	On sale	✓	✓
Olanzapine	N05AH03	Prior to 2005	On sale	✓	✓
Oxypertine	N05AE01	Prior to 2005	On sale	✓	✓

Paliperidone	N05AX13	January 2011	On sale	✓	✓
Perospirone	None	Prior to 2005	On sale	✓	✓
Perphenazine	N05AB03	Prior to 2005	On sale	✓	✓
Pimozide	N05AG02	Prior to 2005	On sale	✓	✓
Prochlorperazine	N05AB04	Prior to 2005	December 2012	✓	✓
Propericyazine	None	Prior to 2005	On sale	✓	✓
Quetiapine	N05AH04	Prior to 2005	On sale	✓	✓
Risperidone	N05AX08	Prior to 2005	On sale	✓	✓
Spiperone	None	Prior to 2005	On sale	✓	✓
Sulpiride	N05AL01	Prior to 2005	On sale	✓	✓
Sultopride	N05AL02	Prior to 2005	On sale	✓	✓
Tiapride	N05AL03	Prior to 2005	On sale	✓	✓
Thioridazine	N05AC02	Prior to 2005	March 2007	✓	✓
Timiperone	None	Prior to 2005	On sale	✓	✓
Trifluoperazine	N05AB06	Prior to 2005	June 2013	✓	✓
Zotepine	N05AX11	Prior to 2005	On sale	✓	✓

Note:

[A] Takeshima M, Enomoto M, Ogasawara M, Kudo M, Itoh Y, Yoshizawa K, et al. Changes in psychotropic polypharmacy and high-potency prescription following policy change: Findings from a large scale Japanese claims database. Psychiatry and clinical neurosciences. 2022.

[B] Takeshima M, Yoshizawa K, Enomoto M, Ogasawara M, Kudo M, Itoh Y, et al. Effects of Japanese policies and novel hypnotics on long-term prescriptions of hypnotics. Psychiatry and clinical neurosciences. 2023;77(5):264-72.

Abbreviation: ATC, the Anatomical Therapeutic Chemical classification 2021
