# Supplementary material for: Association between polypharmacy and the long-term prescription of hypnotics in Japan: a retrospective cross-sectional study
Source: Front Psychiatry. 2024 Dec 9;15:1471457. doi: 10.3389/fpsyt.2024.1471457 (PMC11663738; doi:10.3389/fpsyt.2024.1471457)
Supplement: Supplementary file 3 [file DataSheet3.pdf]

Table S3. Complete version of clinical and demographic characteristics

	Total<br>N=112256	Monotherapy<br>N=76207	Polypharmacy<br>N=36049	p-value	
**Age group**				<0.001**	
20–39	23664 (21.1%)	15366 (20.2%)	8298 (23.0%)		M<P
40–64	77665 (69.2%)	52031 (68.3%)	25634 (71.1%)		M<P
>=65	10927 (9.7%)	8810 (11.6%)	2117 (5.9%)		M>P
**Sex**				<0.001**	
Male	59434 (52.9%)	39804 (52.2%)	19630 (54.5%)		
Female	52822 (47.1%)	36403 (47.8%)	16419 (45.5%)		
**Subscriber**				0.074	
Him/Herself	75794 (67.5%)	51585 (67.7%)	24209 (67.2%)		
Families	36462 (32.5%)	24622 (32.3%)	11840 (32.8%)		
**Hypnotic prescription duration**				<0.001**	
1 month	5341 (4.8%)	4604 (6.0%)	737 (2.0%)		M>P
2–3 months	6894 (6.1%)	5791 (7.6%)	1103 (3.1%)		M>P
4–6 months	8713 (7.8%)	7224 (9.5%)	1489 (4.1%)		M>P
7–9 months	8669 (7.7%)	7004 (9.2%)	1665 (4.6%)		M>P
10–12 months	8697 (7.7%)	6834 (9.0%)	1863 (5.2%)		M>P
13–24 months	73942 (65.9%)	44750 (58.7%)	29192 (81.0%)		M<P
**Class of hypnotics**					
BZ	62736 (55.9%)	33263 (43.6%)	29473 (81.8%)	<0.001**	
BZ hypnotics	57388 (51.1%)	30591 (40.1%)	26797 (74.3%)	<0.001**	
BZ anxiolytics at bedtime	9221 (8.2%)	2672 (3.5%)	6549 (18.2%)	<0.001**	
Z-drug	49527 (44.1%)	31499 (41.3%)	18028 (50.0%)	<0.001**	
MRA	4877 (4.3%)	1888 (2.5%)	2989 (8.3%)	<0.001**	
ORA	17769 (15.8%)	8695 (11.4%)	9074 (25.2%)	<0.001**	
Trazodone	4553 (4.1%)	525 (0.7%)	4028 (11.2%)	<0.001**	
Quetiapine	1841 (1.6%)	323 (0.4%)	1518 (4.2%)	<0.001**	
Hydroxyzine	65 (0.1%)	14 (0%)	51 (0.1%)	<0.001**	

**Hypnotics class combinations**					
BZ only	42357 (37.7%)	33263 (43.6%)	9094 (25.2%)		M>P
Z-drug only	32196 (28.7%)	31499 (41.3%)	697 (1.9%)		M>P
MRA only	1888 (1.7%)	1888 (2.5%)	0 (0%)		M>P
ORA only	8779 (7.8%)	8695 (11.4%)	84 (0.2%)		M>P
Trazodone only	525 (0.5%)	525 (0.7%)	0 (0%)		M>P
Quetiapine only	323 (0.3%)	323 (0.4%)	0 (0%)		M>P
Hydroxyzine only	14 (0%)	14 (0%)	0 (0%)		M>P
BZ+Z-drug	10864 (9.7%)	0 (0%)	10864 (30.1%)		M<P
BZ+MRA	979 (0.9%)	0 (0%)	979 (2.7%)		M<P
BZ+ORA	3754 (3.3%)	0 (0%)	3754 (10.4%)		M<P
BZ+Trazodone	1741 (1.6%)	0 (0%)	1741 (4.8%)		M<P
BZ+Quetiapine	628 (0.6%)	0 (0%)	628 (1.7%)		M<P
BZ+Hydroxyzine	19 (0%)	0 (0%)	19 (0.1%)		M<P
Z-drug+MRA	694 (0.6%)	0 (0%)	694 (1.9%)		M<P
Z-drug+ORA	2674 (2.4%)	0 (0%)	2674 (7.4%)		M<P
Z-drug+Trazodone	738 (0.7%)	0 (0%)	738 (2%)		M<P
Z-drug+Quetiapine	236 (0.2%)	0 (0%)	236 (0.7%)		M<P
Z-drug+Hydroxyzine	13 (0%)	0 (0%)	13 (0%)		M<P
MRA+ORA	570 (0.5%)	0 (0%)	570 (1.6%)		M<P
MRA+Trazodone	58 (0.1%)	0 (0%)	58 (0.2%)		M<P
MRA+Quetiapine	25 (0%)	0 (0%)	25 (0.1%)		M<P
MRA+Hydroxyzine	1 (0%)	0 (0%)	1 (0%)		M<P
ORA+Trazodone	298 (0.3%)	0 (0%)	298 (0.8%)		M<P
ORA+Quetiapine	124 (0.1%)	0 (0%)	124 (0.3%)		M<P
ORA+Hydroxyzine	2 (0%)	0 (0%)	2 (0%)		M<P
Trazodone+Quetiapine	11 (0%)	0 (0%)	11 (0%)		M<P
3 or more	2745 (2.4%)	0 (0%)	2745 (7.6%)		M<P
**Anxiolytics**					
BZ Anxiolytics during the day	32153 (28.6%)	19187 (25.2%)	12966 (36%)	<0.001**	
Hydroxyzine during the day	49 (0%)	24 (0%)	25 (0.1%)	0.005*	
Tandospirone	918 (0.8%)	573 (0.8%)	345 (1%)	<0.001**	
**Antidepressant**				<0.001**	
0	68759 (61.3%)	50931 (66.8%)	17828 (49.5%)		M>P

1	32287 (28.8%)	19522 (25.6%)	12765 (35.4%)		M<P
≥2	11210 (10%)	5754 (7.6%)	5456 (15.1%)		M<P
**Antipsychotics**				<0.001**	
0	91855 (81.8%)	65578 (86.1%)	26277 (72.9%)		M>P
1	15215 (13.6%)	8195 (10.8%)	7020 (19.5%)		M<P
≥2	5186 (4.6%)	2434 (3.2%)	2752 (7.6%)		M<P
**Sleep disorders**					
SRBD	3118 (2.8%)	2090 (2.7%)	1028 (2.9%)	0.30	
CRSWD	94 (0.1%)	33 (0%)	61 (0.2%)	<0.001**	
**Psychiatric disorders**					
Substance use disorder				<0.001**	
None	110393 (98.3%)	75306 (98.8%)	35087 (97.3%)		M>P
Alcohol-related disorders	1434 (1.3%)	672 (0.9%)	762 (2.1%)		M<P
Opioid-related disorders	2 (0%)	1 (0%)	1 (0%)		M=P
Cannabis-related disorders	1 (0%)	1 (0%)	0 (0%)		M=P
Sedative, hypnotic, or anxiolytic-related disorders	10 (0%)	4 (0%)	6 (0%)		M=P
Other stimulant-related disorders including caffeine	15 (0%)	4 (0%)	11 (0%)		M<P
Nicotine dependence	286 (0.3%)	172 (0.2%)	114 (0.3%)		M<P
Multiple drug use and other psychoactive substance-related disorders	115 (0.1%)	47 (0.1%)	68 (0.2%)		M<P
Schizophrenia	20302 (18.1%)	10141 (13.3%)	10161 (28.2%)	<0.001**	
Depressive disorders	56305 (50.2%)	32149 (42.2%)	24156 (67%)	<0.001**	
Bipolar disorders	13443 (12%)	6750 (8.9%)	6693 (18.6%)	<0.001**	
Anxiety disorders	22731 (20.2%)	13840 (18.2%)	8891 (24.7%)	<0.001**	
Reaction to severe stress, and adjustment disorders	5807 (5.2%)	3710 (4.9%)	2097 (5.8%)	<0.001**	
Personality disorder	419 (0.4%)	203 (0.3%)	216 (0.6%)	<0.001**	
**Physical disorders**					
Cardiovascular_diseasess	11337 (10.1%)	8215 (10.8%)	3122 (8.7%)	<0.001**	
Diabetes	16032 (14.3%)	11379 (14.9%)	4653 (12.9%)	<0.001**	

Chronic kidney diseases	1836 (1.6%)	1332 (1.7%)	504 (1.4%)	<0.001**
COPD	852 (0.8%)	614 (0.8%)	238 (0.7%)	0.009*
Rheumatoid arthritis	1776 (1.6%)	1249 (1.6%)	527 (1.5%)	0.026*
Chronic pain	3772 (3.4%)	2461 (3.2%)	1311 (3.6%)	<0.001**
Cancer	268 (0.2%)	212 (0.3%)	56 (0.2%)	<0.001**
Neurodegenerative_diseases	2989 (2.7%)	1612 (2.1%)	1377 (3.8%)	<0.001**
Cerebrovascular_diseases	4602 (4.1%)	3446 (4.5%)	1156 (3.2%)	<0.001**
Traumatic brain injury	108 (0.1%)	75 (0.1%)	33 (0.1%)	0.73
Multiple sclerosis	114 (0.1%)	84 (0.1%)	30 (0.1%)	0.19

Note: Values are presented as numbers (%). P-values with significant results (<0.05) are labeled with an asterisk, and those with significant results (<0.001**) are labeled with a double asterisk.

M>P indicates a significantly larger proportion of patients in the polypharmacy group than that in the monotherapy group for that item based on post-hoc analysis.

M<P indicates a significantly larger proportion of patients in the monotherapy group than that in the polypharmacy group for that item based on post-hoc analysis.

Abbreviations: BZ, benzodiazepine; CI, confidence interval; COPD, chronic obstructive pulmonary disease; CRSWD, circadian rhythm sleep-wake disorder; MRA, melatonin receptor agonist; OR, odds ratio; ORA, orexin receptor antagonist; SRBD, sleep related breathing disorders
