# Supplementary material for: Association between polypharmacy and the long-term prescription of hypnotics in Japan: a retrospective cross-sectional study
Source: Front Psychiatry. 2024 Dec 9;15:1471457. doi: 10.3389/fpsyt.2024.1471457 (PMC11663738; doi:10.3389/fpsyt.2024.1471457)
Supplement: Supplementary file 4 [file DataSheet4.pdf]

Table S4. Sensitivity analysis

	Adjusted OR (95%CI)	p-value
Patients not prescribed psychotropic other than hypnotics [a] (N=47,953)		
1 month	1 [Reference]	
2–3 months	1.02 (0.87–1.20)	0.81
4–6 months	1.04 (0.89–1.21)	0.63
7–9 months	1.23 (1.06–1.43)	0.006
10–12 months	1.52 (1.32–1.76)	<0.001
13–24 months	2.95 (2.61–3.34)	<0.001
Patients without physical disorder [b] (N=69,316)		
1 month		
2–3 months	1.15 (1.03–1.29)	0.015
4–6 months	1.24 (1.11–1.39)	<0.001
7–9 months	1.51 (1.35–1.68)	<0.001
10–12 months	1.70 (1.52–1.89)	<0.001
13–24 months	3.49 (3.19–3.83)	<0.001
Patients without psychiatric disorder [c] (N=81,088)		
1 month		
2–3 months	0.94 (0.78–1.13)	0.52
4–6 months	0.90 (0.75–1.08)	0.25
7–9 months	1.06 (0.89–1.27)	0.49
10–12 months	1.42 (1.20–1.68)	<0.001
13–24 months	2.47 (2.15–2.84)	<0.001
Patients not prescribed psychotropic other than hypnotics (N=52,667)		
1 month	1 [Reference]	
2–3 months	1.02 (0.87–1.20)	0.81
4–6 months	1.04 (0.89–1.21)	0.63
7–9 months	1.23 (1.06–1.43)	0.006
10–12 months	1.52 (1.32–1.76)	<0.001
13–24 months	2.95 (2.61–3.34)	<0.001

Note: P-values with significant results (<0.05) are labeled with an asterisk, and those with significant results (<0.001) are labeled with a double asterisk. The larger the odds ratio, the stronger the positive

association with hypnotic polypharmacy.

[a] Adjusted for age groups (20–39, 40–64, and 65–74 years); sex; type of subscriber (employees and their family members); each sleep disorder, each psychiatric disorder, and each physical disorder.

[b] Adjusted for age groups (20–39, 40–64, and 65–74 years); sex; type of subscriber (employees and their family members); the number of concomitant antidepressants, antipsychotics, benzodiazepine anxiolytics during the day, hydroxyzine during the day, and tandspirone (0, 1, 2 or more); each sleep disorder, and each physical disorder.

[c] Adjusted for age groups (20–39, 40–64, and 65–74 years); sex; type of subscriber (employees and their family members); hypnotic prescription duration (1, 2–3, 4–6, 7–9, 10–12, and 13–24 months); the number of concomitant antidepressants, antipsychotics, benzodiazepine anxiolytics during the day, hydroxyzine during the day, and tandspirone (0, 1, 2 or more); each sleep disorder, and each psychiatric disorder.

Abbreviations: CI, confidence interval; OR, odds ratio.
