# Supplementary material for: Association between polypharmacy and the long-term prescription of hypnotics in Japan: a retrospective cross-sectional study
Source: Front Psychiatry. 2024 Dec 9;15:1471457. doi: 10.3389/fpsyt.2024.1471457 (PMC11663738; doi:10.3389/fpsyt.2024.1471457)
Supplement: Supplementary file 5 [file DataSheet5.pdf]

Table S5. Complete version of logistic regression analysis model 2 examining the association between hypnotic polypharmacy and individual antidepressants/antipsychotics

	Adjusted OR (95% CI)	p-value
**Antidepressants**		
None	1 [Reference]	
Mianserin	1.64 (1.23–2.19)	<0.001**
Lofepramine	1.49 (0.67–3.33)	0.33
Amoxapine	1.41 (1.18–1.69)	<0.001**
Dosulepin	1.25 (0.73–2.13)	0.41
Trazodone	1.24 (1.09–1.40)	<0.001**
Setiptiline	1.11 (0.58–2.14)	0.75
Imipramine	1.09 (0.80–1.48)	0.58
Mirtazapine	1.08 (1.00–1.17)	0.058
Milnacipran	1.06 (0.86–1.31)	0.56
Nortriptyline	1.04 (0.73–1.49)	0.81
Duloxetine	1.04 (0.98–1.11)	0.22
Vortioxetine	1.03 (0.95–1.19)	0.65
Maprotiline	1.02 (0.74–1.40)	0.92
Venlafaxine	0.99 (0.89–1.11)	0.87
Sulpiride	0.95 (0.88–1.03)	0.23
Amitriptyline	0.95 (0.80–1.12)	0.53
Fluvoxamine	0.92 (0.82–1.03)	0.17
Sertraline	0.90 (0.84–0.96)	0.002*
Clomipramine	0.79 (0.63–0.98)	0.033*
Escitalopram	0.87 (0.81–0.93)	<0.001**
Paroxetine	0.82 (0.76–0.89)	<0.001**
Trimipramine	0.72 (0.11–4.65)	0.73
2 or more	1.21 (1.15–1.27)	<0.001**
**Antipsychotics**		
None	1 [Reference]	
Floropipamide	6.37 (0.53–76.4)	0.14
Oxypertine	3.34 (0.27–40.7)	0.35
Tiapride	2.33 (1.29–4.21)	0.005*

Levomepromazine	2.06 (1.79–2.37)	<0.001**
Chlorpromazine	1.76 (1.49–2.07)	<0.001**
Pimozide	1.62 (0.13–19.7)	0.70
Sulpiride	1.54 (1.11–2.15)	0.01*
Zotepine	1.46 (0.86–2.50)	0.17
Prochlorperazine	1.28 (0.81–2.02)	0.29
Lurasidone	1.23 (1.01–1.50)	0.04*
Perospirone	1.21 (0.94–1.55)	0.14
Asenapine	1.20 (0.78–1.84)	0.42
Blonanserin	1.14 (0.91–1.42)	0.25
Quetiapine	1.08 (0.97–1.20)	0.16
Haloperidol	1.07 (0.79–1.46)	0.66
Perphenazine	1.03 (0.76–1.40)	0.83
Risperidone	1.01 (0.90–1.14)	0.81
Aripiprazole	1.00 (0.94–1.06)	0.92
Clocapramine	0.97 (0.23–4.03)	0.96
Brexpiprazole	0.96 (0.81–1.13)	0.59
Blonanserin (tape)	0.92 (0.27–3.11)	0.90
Bromperidol	0.89 (0.47–1.69)	0.72
Olanzapine	0.85 (0.77–0.94)	0.001*
Paliperidone	0.79 (0.53–1.16)	0.23
Fluphenazine	0.78 (0.21–2.85)	0.71
Sultopride	0.78 (0.17–3.62)	0.76
Clozapine	0.76 (0.31–1.88)	0.55
Timiperone	0.68 (0.053–8.72)	0.77
2 or more	1.35 (1.25–1.45)	<0.001**

Note: P-values with significant results (<0.05) are labeled with an asterisk (*), and those with significant results (<0.001) are labeled with a double asterisk (**). The larger the odds ratio, the stronger the positive association with hypnotic polypharmacy.

Adjusted for age groups (20–39, 40–64, and 65–74 years); sex; type of subscriber (employees and their family members); hypnotic prescription duration (1, 2–3, 4–6, 7–9, 10–12, and 13–24 months); the number of concomitant antidepressants, antipsychotics, benzodiazepine anxiolytics during the day, hydroxyzine during the day, and tandospirone (0, 1, 2 or more); each sleep disorder, each psychiatric disorder, and each physical disorder.

The odds ratio could not be calculated because there were no patients who is prescribed nemonapride and mosapramine in the hypnotic monotherapy group.

Abbreviations: CI, confidence interval; OR, odds ratio.
